# Supplementary material for: Cyclometallated Palladium(II) Complexes: An Approach to the First Dinuclear Bis(iminophosphorane)phosphane-[C,N,S] Metallacycle
Source: Molecules. 2022 Oct 19;27(20):7043. doi: 10.3390/molecules27207043 (PMC9607290; doi:10.3390/molecules27207043)
Supplement: Supplementary file 1 [file molecules-27-07043-s001.zip › molecules-1960323-supplementary.pdf]

Supporting Information

Crystal structure analysis

Three-dimensional, X-ray data were collected on a Bruker Kappa-APEX II; Siemens Smart CCD diffractometer by the ω scan method using graphite-monochromated Mo−Kα radiation. All the measured reflections were corrected for Lorentz and polarisation effects and for absorption by semi-empirical methods based on symmetry-equivalent and repeated reflections. The structures were solved by direct methods and refined by full matrix least squares on F^2. Hydrogen atoms were included in calculated positions. Refinement converged at a final R_1 = 0.0638 and wR_2 = 0.1549 (compound **3b**) and R_1 = 0.0508 and wR_2 = 0.1189 (compound **5**) with allowance for thermal anisotropy of all non-hydrogen atoms. The structure solution and refinement were carried out using the programs package SHELX-97[1] and OLEX2.[2]

Table S1 Crystal data and structure refinement for **3c**

Identification code	**3c**	
Empirical formula	$C_{41} H_{38} Cl_2 N_2 P_2 Pd_2 S_2$, 2 $C_3 H_6 O$	
Formula weight	1084.65	
Temperature	100(2) K	
Wavelength	0.71073 Å	
Crystal system	Orthorhombic	
Space group	Pnma	
Unit cell dimensions	*a* = 19.8757(18) Å	α = 90°.
	b = 24.154(3) Å	β = 90°.
	c = 9.7026(10) Å	γ = 90°.
Volume	4658.0(8) $Å^3$	
Z	4	
Density (calculated)	1.547 Mg/m^3	
Absorption coefficient	1.085 mm^{-1}	
F(000)	2200	
Crystal size	0.180 x 0.050 x 0.030 mm^3	
Theta range for data collection	1.686 to 26.414°.	
Index ranges	-24<=h<=23, -30<=k<=28, -12<=l<=9	
Reflections collected	34838	
Independent reflections	4891 [R(int) = 0.1183]	
Completeness to theta = 25.242°	100.0 %	
Refinement method	Full-matrix least-squares on F^2	
Data / restraints / parameters	4891 / 0 / 281	
Goodness-of-fit on F^2	1.058	

Final R indices [I>2sigma(I)]	R_1 = 0.0638, wR_2 = 0.1549
R indices (all data)	R_1 = 0.1262, wR_2 = 0.1841
Largest diff. peak and hole	2.047 and -1.063 e.Å^{-3}

Table S2 Selected bond lengths [Å] for **3c**

Pd(1)-C(1)	1.962(8)
Pd(1)-N(1)	2.039(6)
Pd(1)-Cl(1)	2.316(2)
Pd(1)-S(1)	2.358(2)
S(1)-C(1)	1.764(7)
S(1)-C(7)	1.804(9)
P(3)-N(1)	1.616(7)
P(3)-C(14)	1.787(7)
P(3)-C(20)	1.810(7)
P(3)-C(8)	1.822(8)

Table S3 Selected angles [°] for **3c**

C(1)-Pd(1)-N(1)	88.5(3)
C(1)-Pd(1)-Cl(1)	94.9(2)
N(1)-Pd(1)-Cl(1)	175.0(2)
C(1)-Pd(1)-S(1)	173.2(2)
N(1)-Pd(1)-S(1)	85.0(2)
Cl(1)-Pd(1)-S(1)	91.77(7)
C(14)-S(1)-C(13)	102.7(4)
C(14)-S(1)-Pd(1)	97.1(3)
C(13)-S(1)-Pd(1)	103.9(3)
N(1)-P(3)-C(8)	114.2(3)
N(1)-P(3)-C(20)	116.6(3)
C(8)-P(3)-C(20)	106.9(3)
N(1)-P(3)-C(2)	101.2(3)

C(8)-P(3)-C(2)	108.7(3)
C(20)-P(3)-C(2)	108.8(3)

Table S4 Crystal data and structure refinement for **5**

Identification code	**5**	
Empirical formula	$C_{39} H_{36} N_2 P_2 Pd S_2$, 2 F_6 P, 2 C H Cl_3	
Formula weight	1293.91	
Temperature	100(2) K	
Wavelength	0.71073 Å	
Crystal system	Monoclinic	
Space group	$P2_1/n$	
Unit cell dimensions	*a* = 20.7967(10) Å	α = 90°.
	b = 8.8579(4) Å	β = 99.064(3)°.
	c = 27.2320(13) Å	γ = 90°.
Volume	4953.9(4) Å3	
Z	4	
Density (calculated)	1.735 Mg/m^3	
Absorption coefficient	0.992 mm^{-1}	
F(000)	2587	
Crystal size	0.400 x 0.170 x 0.150 mm^3	
Theta range for data collection	2.50 to 30.51°.	
Index ranges	-27<=h<=29, -12<=k<=12, -38<=l<=38	
Reflections collected	244322	
Independent reflections	14531 [R(*int*) = 0.0639]	
Completeness to theta = 25.242°	94.30 %	
Refinement method	Full-matrix least-squares on F^2	
Data / restraints / parameters	14531 / 8 / 626	
Goodness-of-fit on F2	1.029	
Final R indices [I>2sigma(I)]	R_1 = 0.0444, wR_2 = 0.0925	
R indices (all data)	R_1 = 0.0636, wR_2 = 0.1018	
Largest diff. peak and hole	3.276 and -1.112 e.Å^{-3}	

Table S5 Selected bond lengths [Å] for **5**

Pd(1)-N(1)	2.074(2)
Pd(1)-N(2)	2.079(2)
Pd(1)-S(1)	2.266(6)
Pd(1)-S(2)	2.268(6)
P(1)-N(1)	1.616(2)
P(2)-N(2)	1.619(2)
S(1)-C(6)	1.772(3)
S(1)-C(7)	1.802(3)
S(2)-C(13)	1.773(3)
S(2)-C(14)	1.805(3)

Table S6 Selected angles [°] for **5**

N(2)-Pd(1)-N(1)	100.6(8)
N(2)-Pd(1)-S(2)	84.7(6)
N(1)-Pd(1)-S(1)	84.5(6)
N(1)-Pd(1)-S(2)	173.7(6)
N(2)-Pd(1)-S(1)	172.1(6)
S(1)-Pd(1)-S(2)	89.9(2)
N(1)-P(1)-C(15)	107.8(1)
N(1)-P(1)-C(16)	112.2(1)
N(1)-P(1)-C(22)	113.3(3)
C(15)-P(1)-C(16)	104.1(2)
C(15)-P(1)-C(22)	108.2(2)
C(22)-P(1)-C(16)	110.6(3)
C(6)-S(1)-Pd(1)	99.3(9)
C(7)-S(1)-Pd(1)	107.1(1)
C(6)-S(1)-C(7)	101.1(3)
N(2)-P(2)-C(15)	107.3(1)

N(2)-P(2)-C(28)	111.8(1)
N(2)-P(2)-C(34)	113.7(2)
C(15)-P(2)-C(28)	104.4(2)
C(15)-P(2)-C(34)	109.3(1)
C(34)-P(2)-C(28)	109.8(2)
C(13)-S(2)-Pd(1)	99.0(9)
C(14)-S(2)-Pd(1)	107.1(1)
C(13)-S(2)-C(14)	101.0(1)

[1] Sheldrick, G. M.; *Acta Crystallogr. A* **2008**, *64*, 112-122, doi.org/10.1107/S010876730704393.

[2] Dolomanov, O. V.; Bourhis, L. J.; Gildea, R. J.; Howard, J. A.; Puschmann, H.; *J. Appl. Crystallogr.* **2011**, *44*, 339-341, doi.org/10.1107/S0021889808042726.

NMR SPECTRA

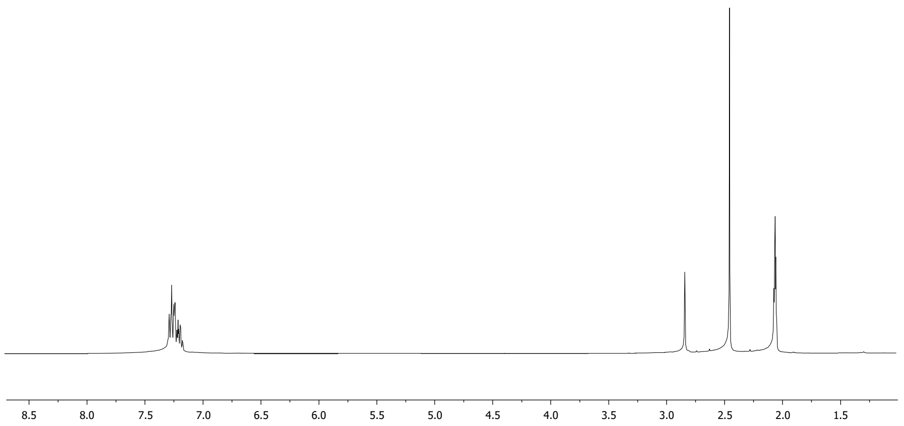

^{1}H NMR spectrum for **1.**

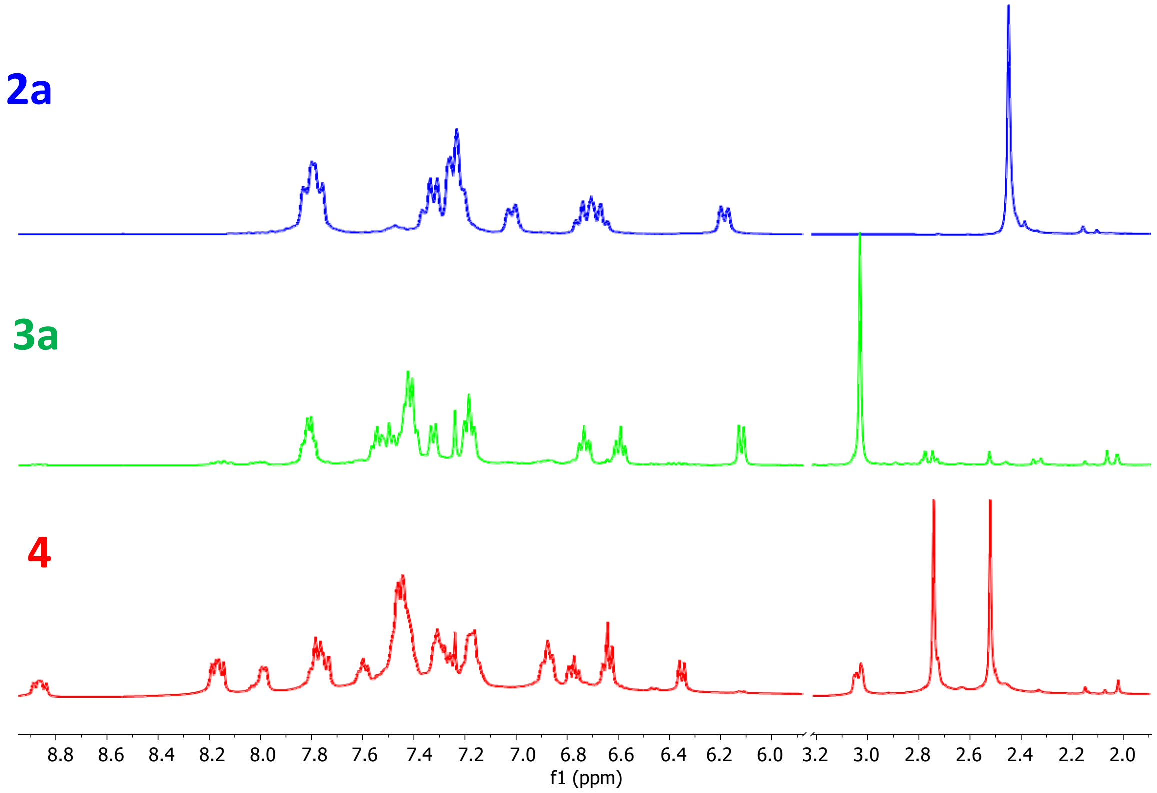

^{1}H NMR spectra for **2a, 3a** and **4.**

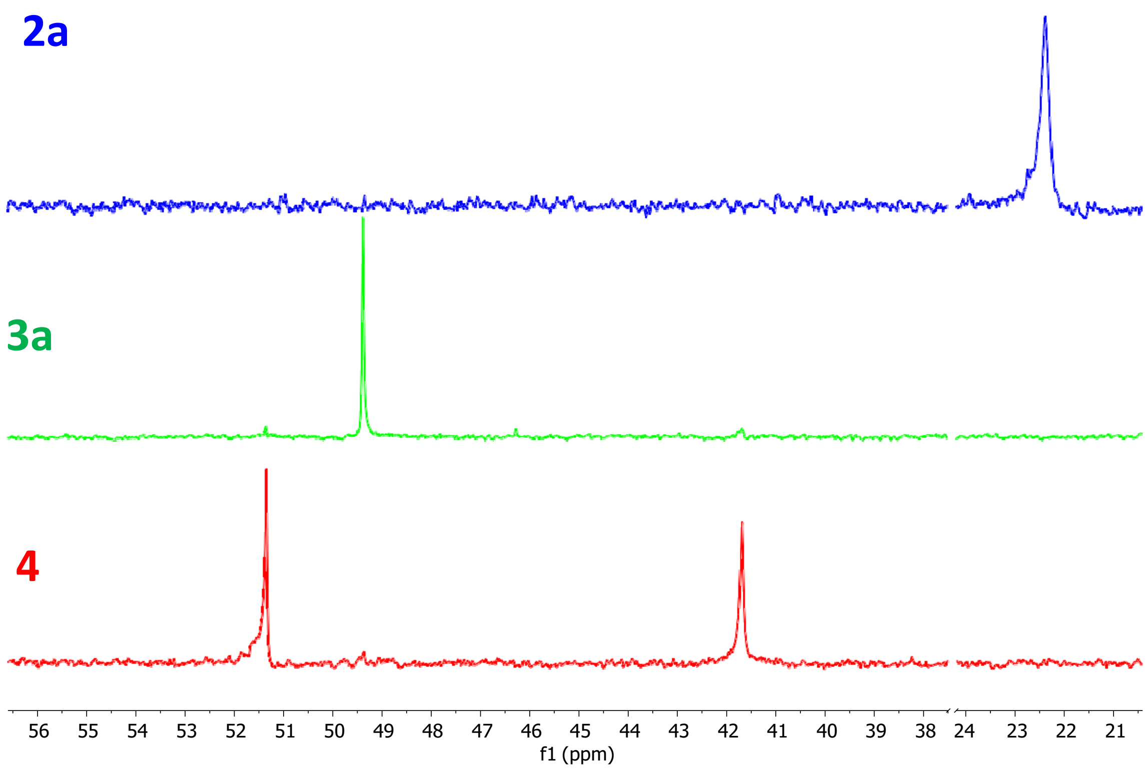

^{31}P-{^{1}H} NMR spectrum for **2a, 3a** and **4.**

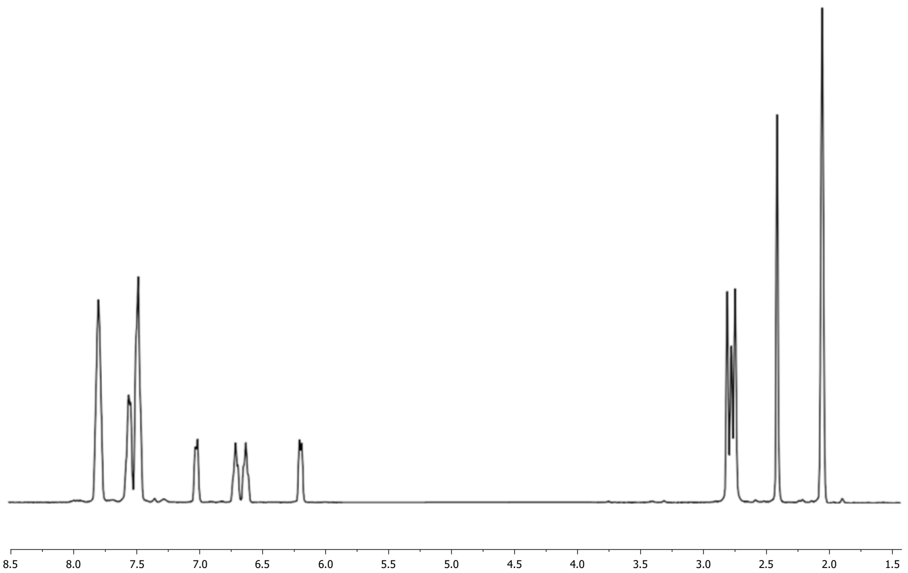

^{1}H NMR spectrum for **2b.**

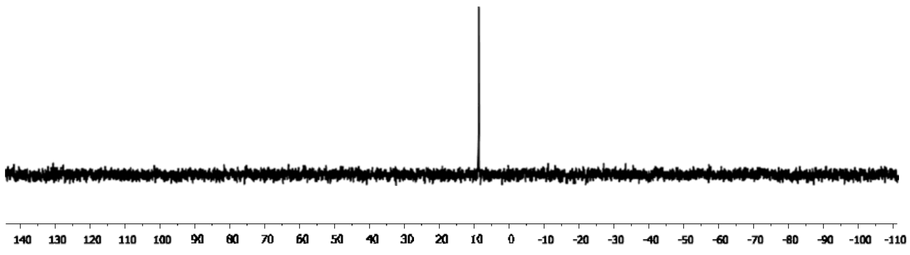

$^{31}P-\{^1H\}$ NMR spectrum for **2b.**

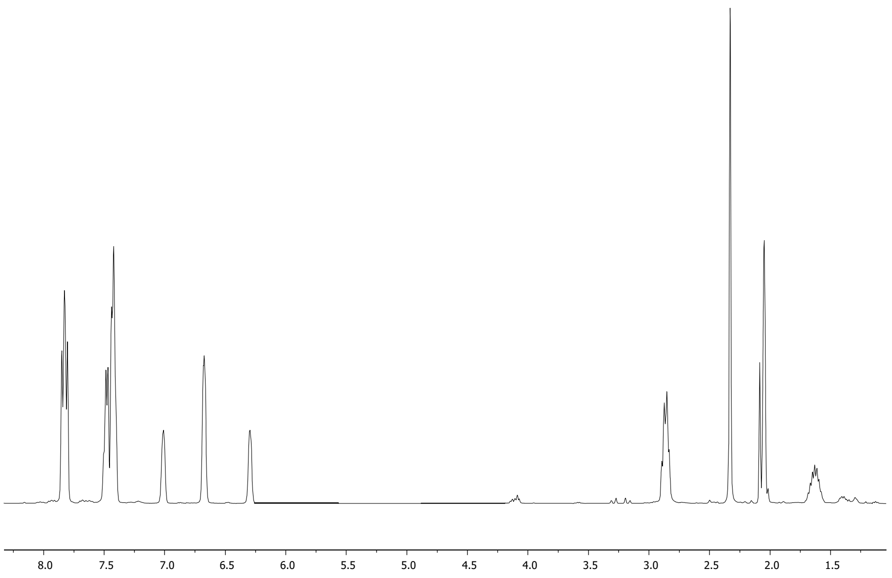

^1H NMR spectrum for **2c.**

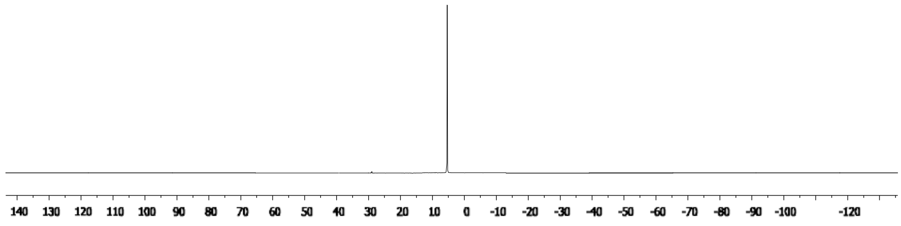

$^{31}P-\{^1H\}$ NMR spectrum for **2c.**

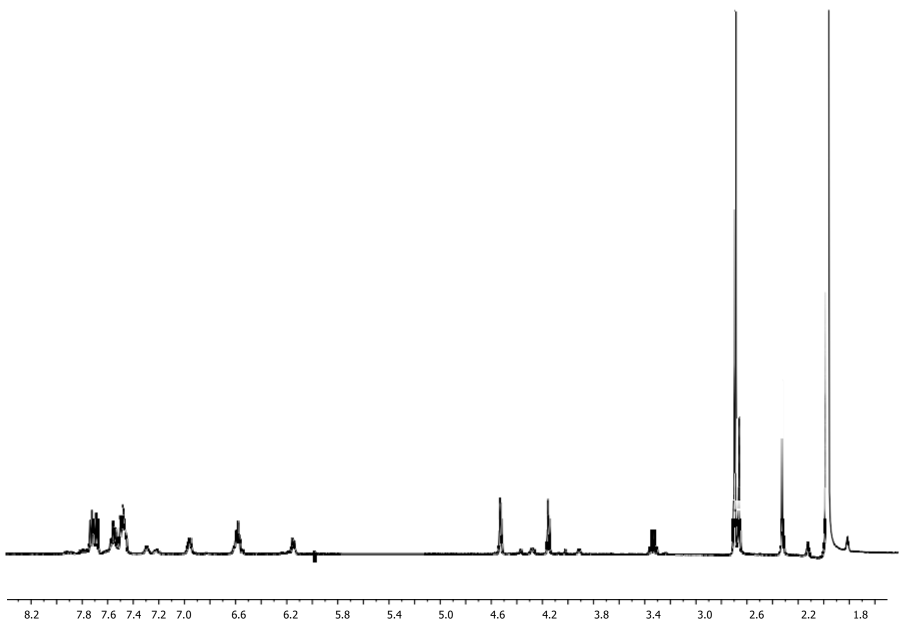

^1H NMR spectrum for **2d.**

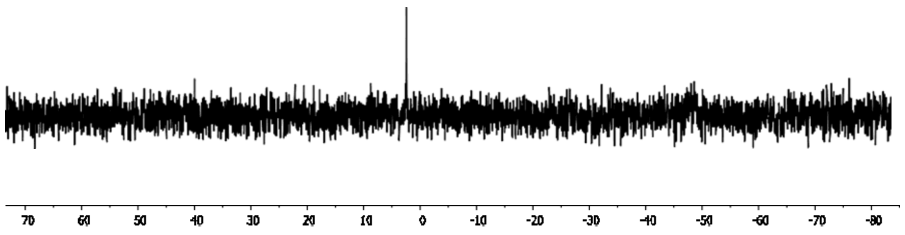

^{31}P-{^1H} NMR spectrum for **2d.**

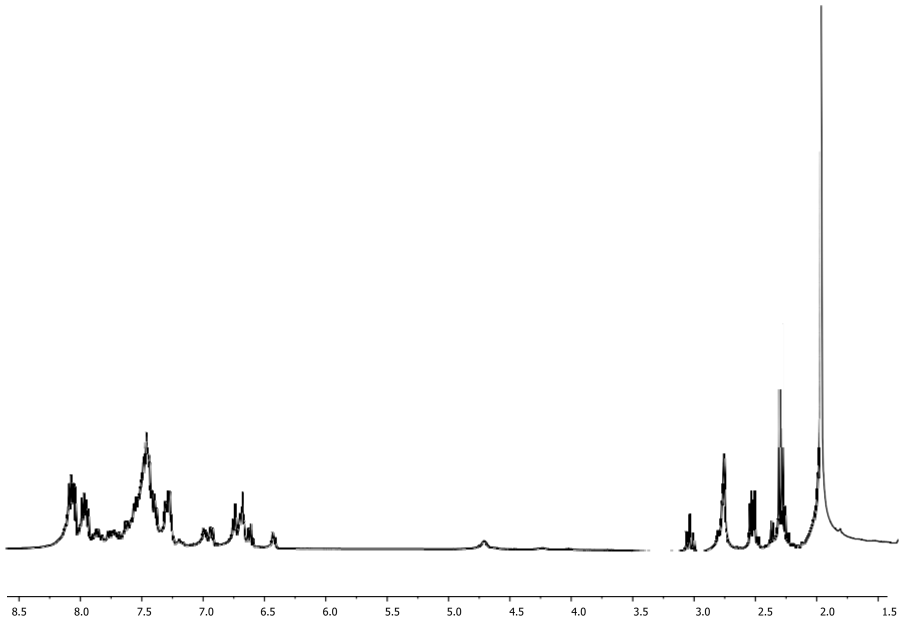

^1H NMR spectrum for **2e.**

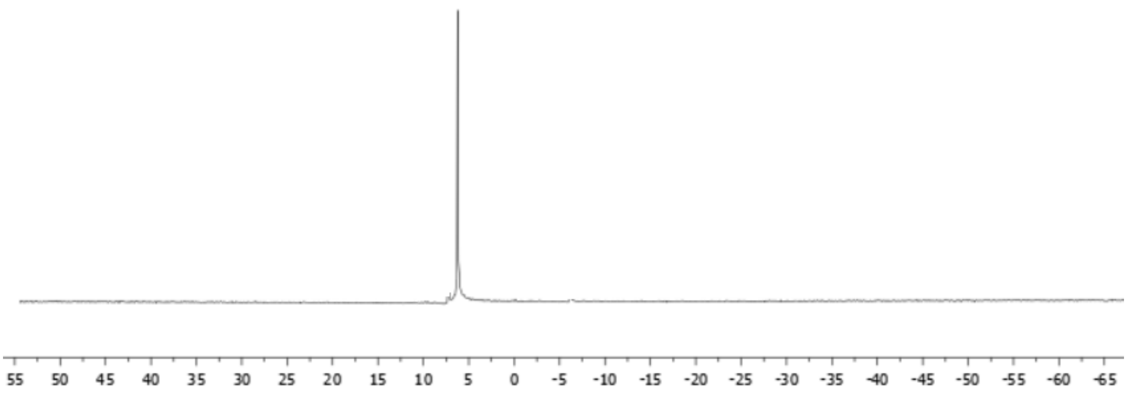

^{31}P-{^{1}H} NMR spectrum for **2e.**

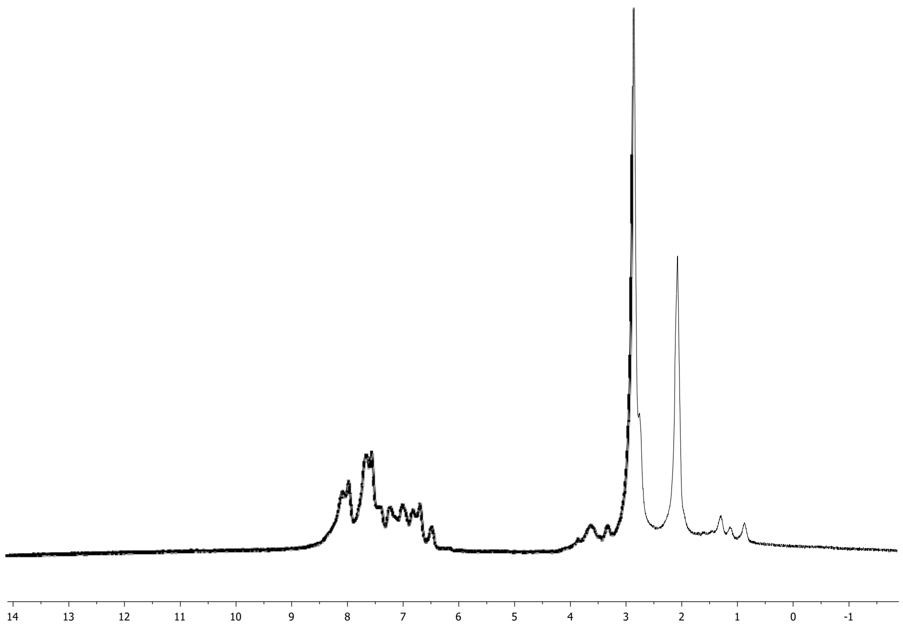

^{1}H NMR spectrum for **3b.**

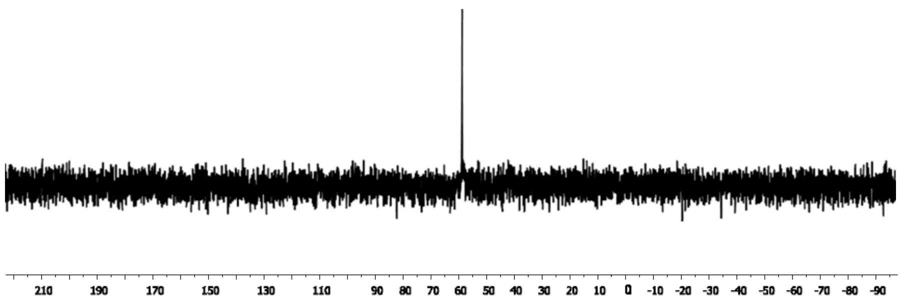

^{31}P-{^{1}H} NMR spectrum for **3b.**

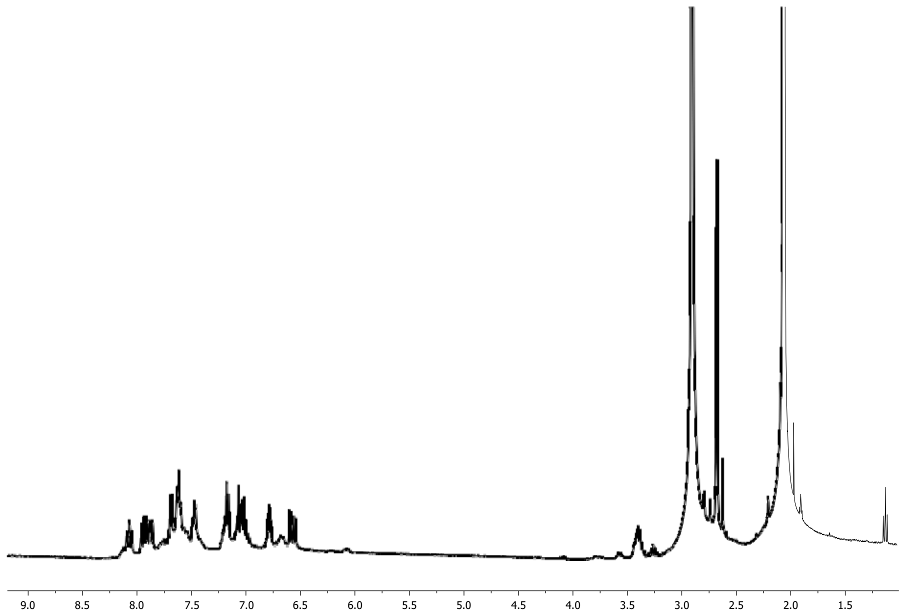

^{1}H NMR spectrum for **3c.**

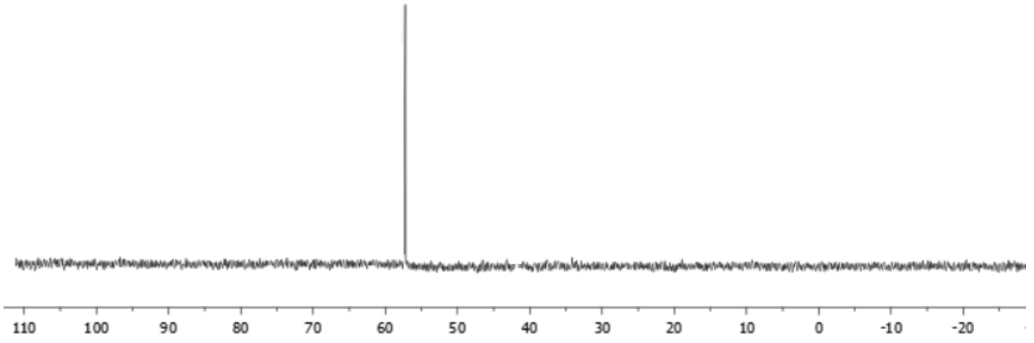

^{31}P-{^{1}H} NMR spectrum for **3c.**

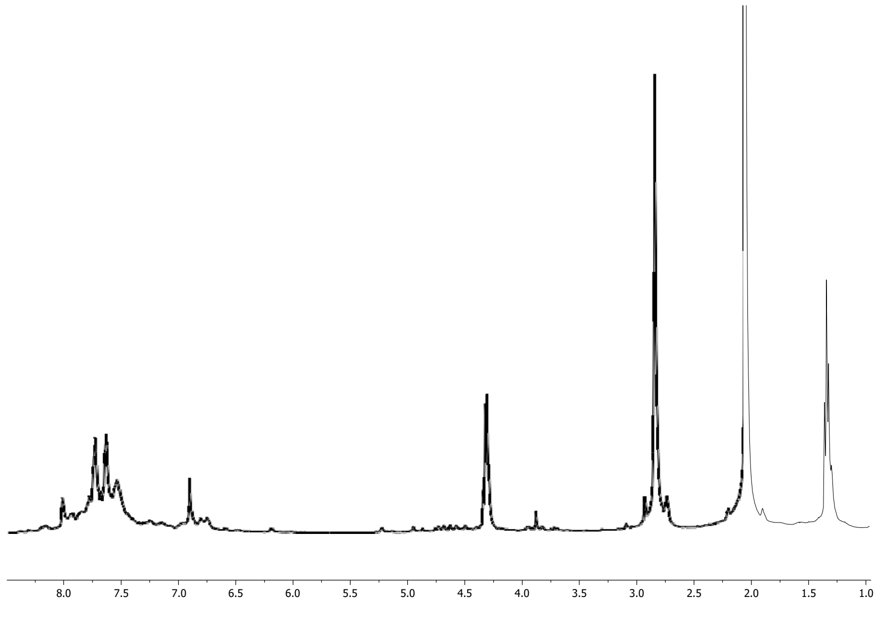

^{1}H NMR spectrum for **3d.**

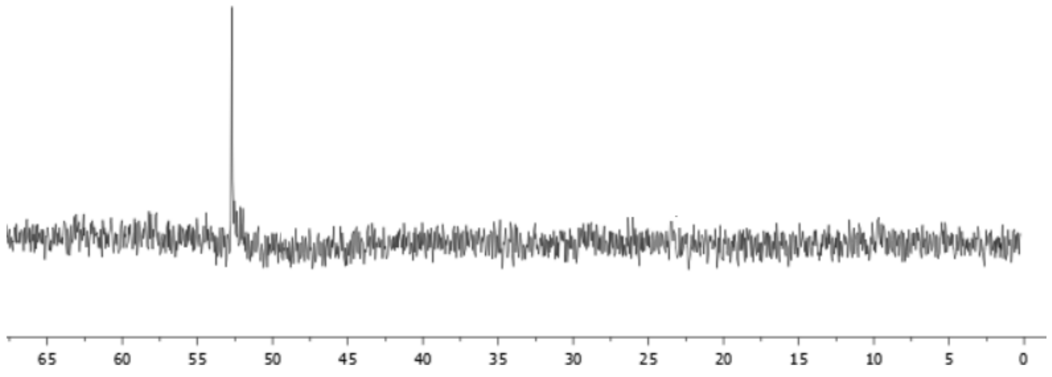

^{31}P-{^1H} NMR spectrum for **3d.**

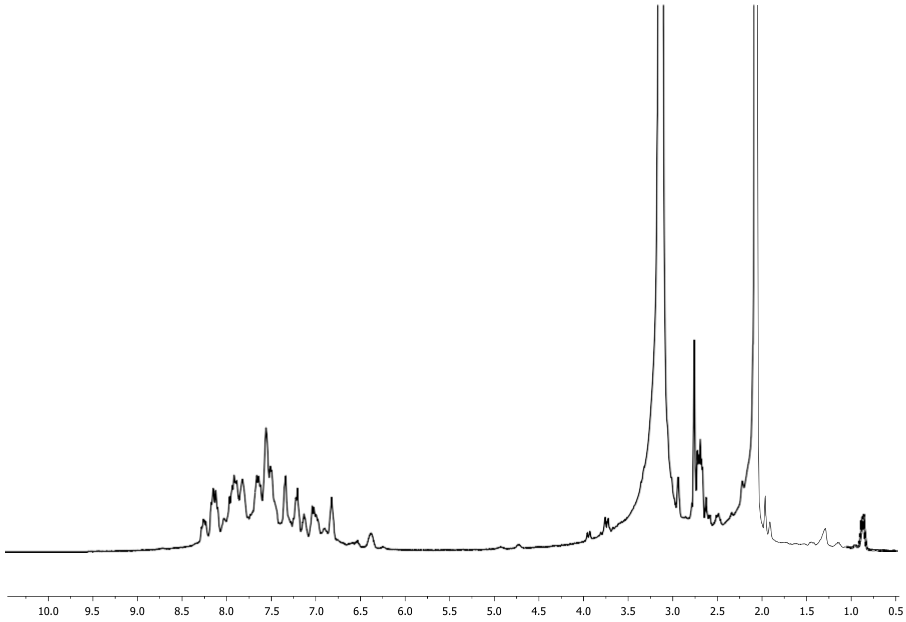

^1H NMR spectrum for **3e.**

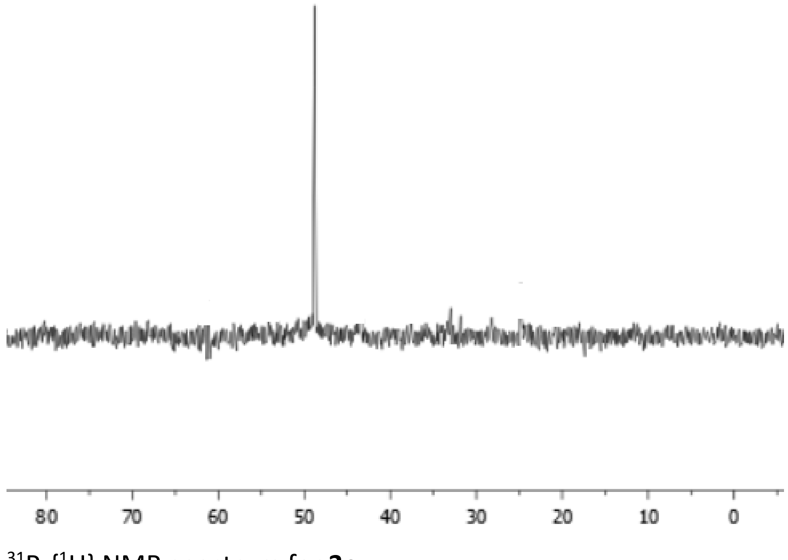

^{31}P-{^1H} NMR spectrum for **3e.**

IR SPECTRA

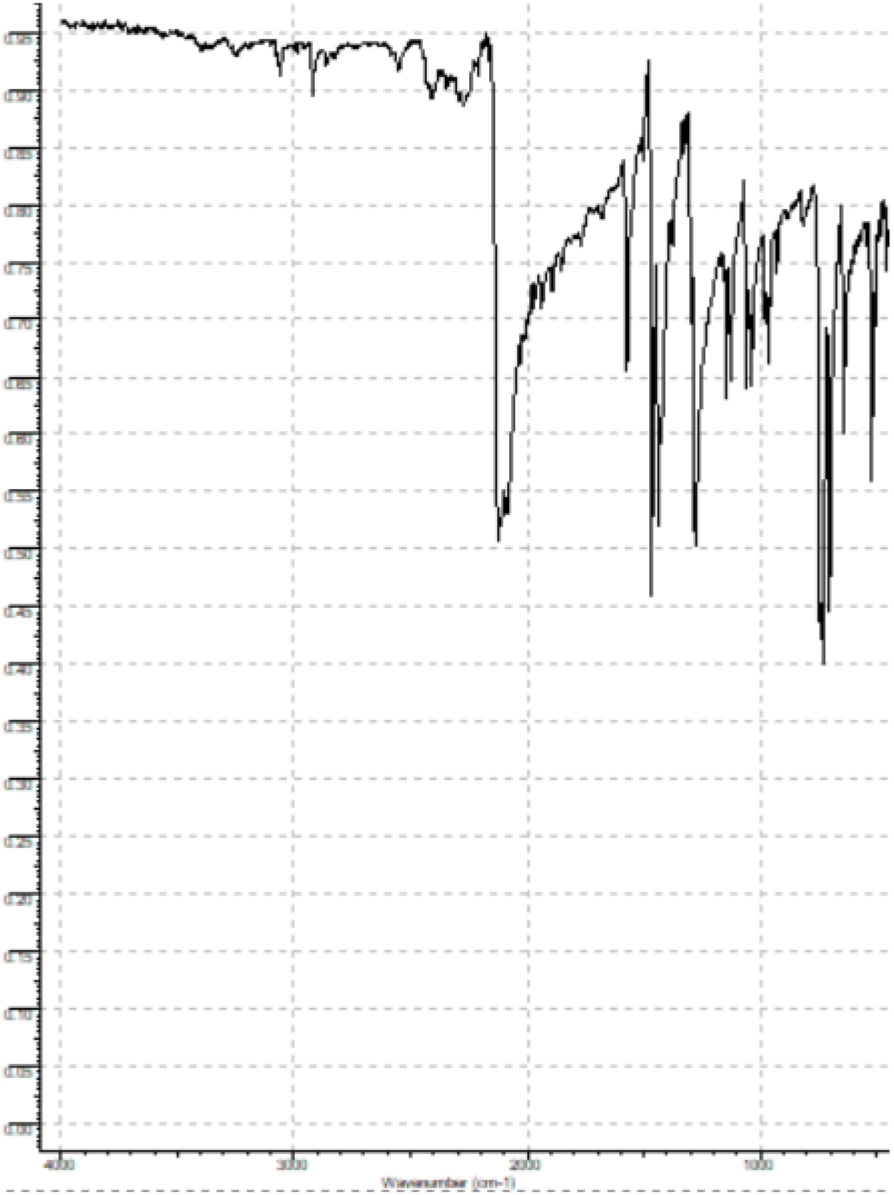

IR spectrum for **1**

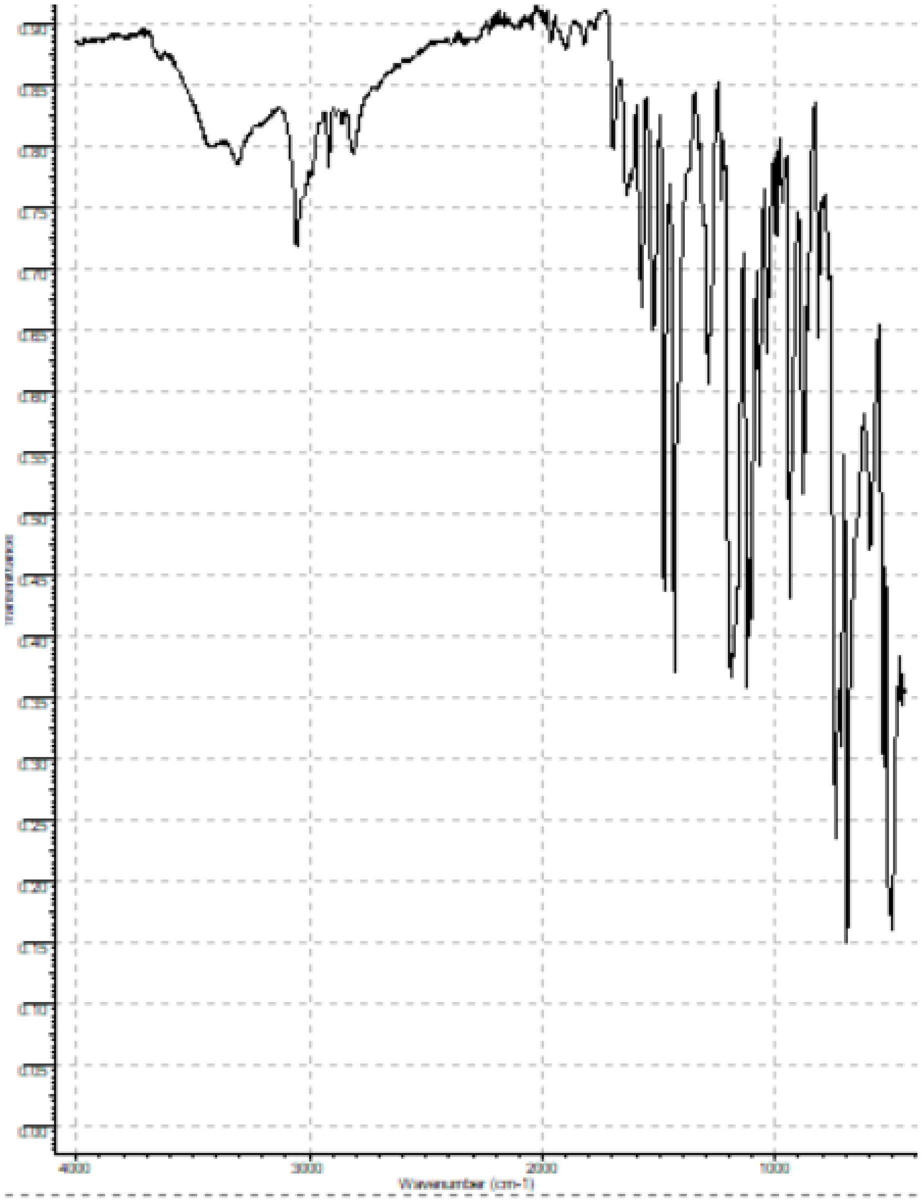

IR spectrum for **2a**

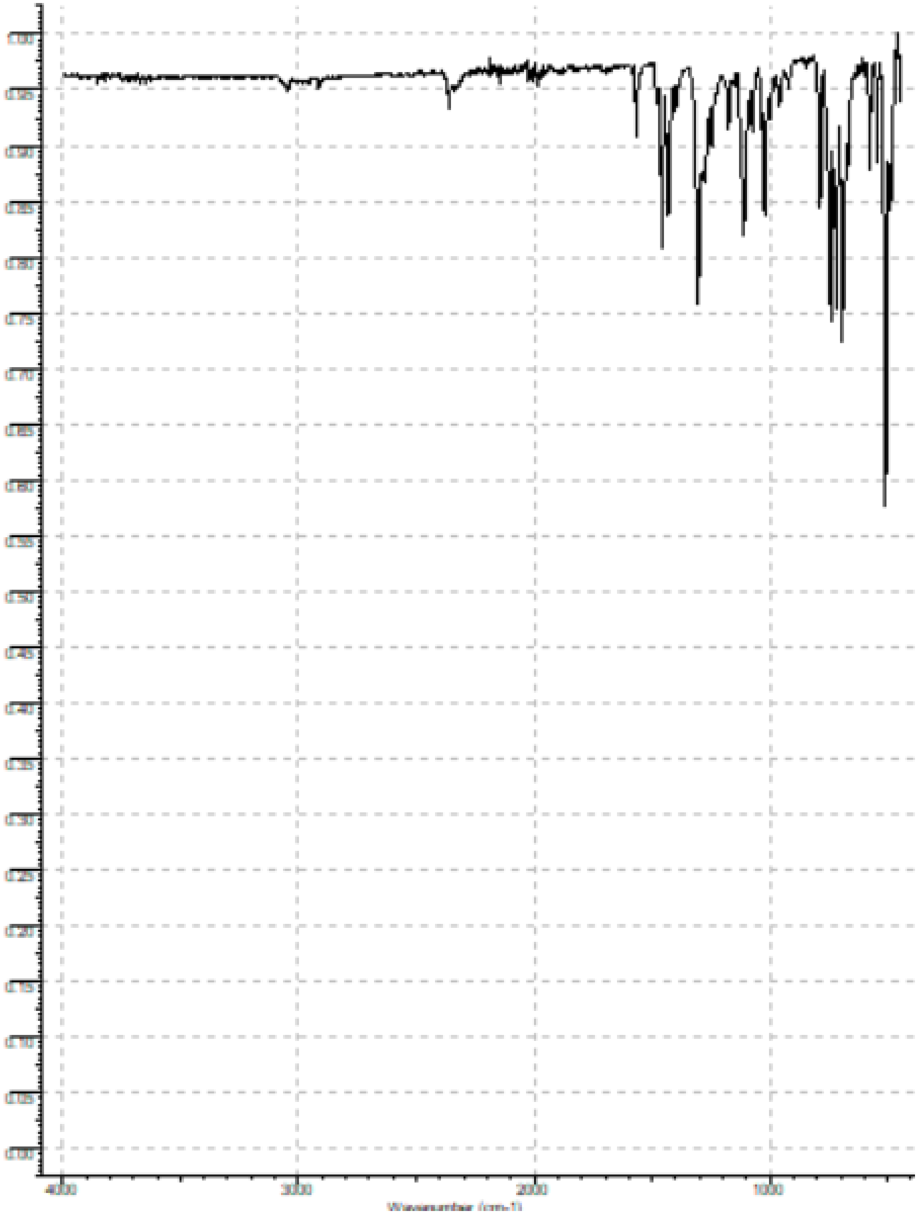

IR spectrum for **2b**

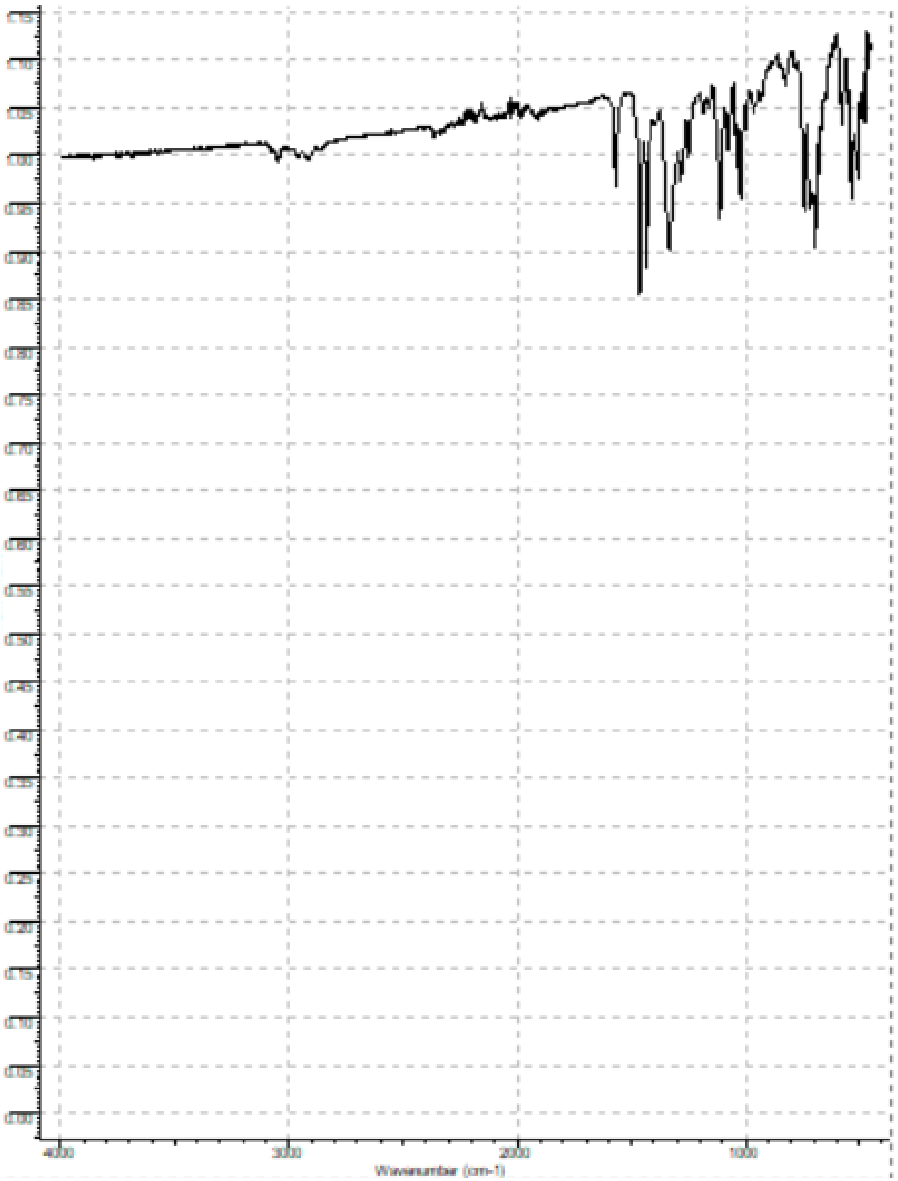

IR spectrum for **2c**

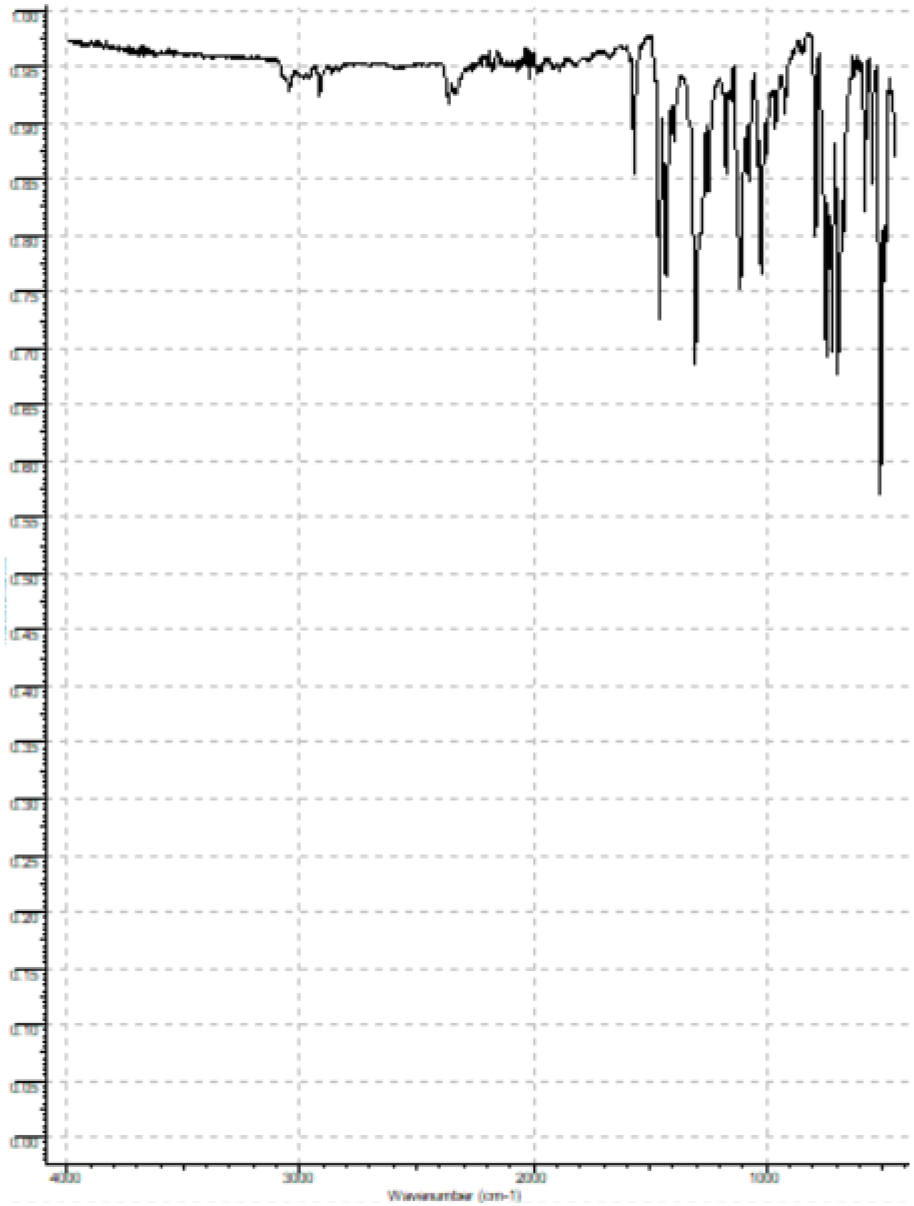

IR spectrum for **2d**

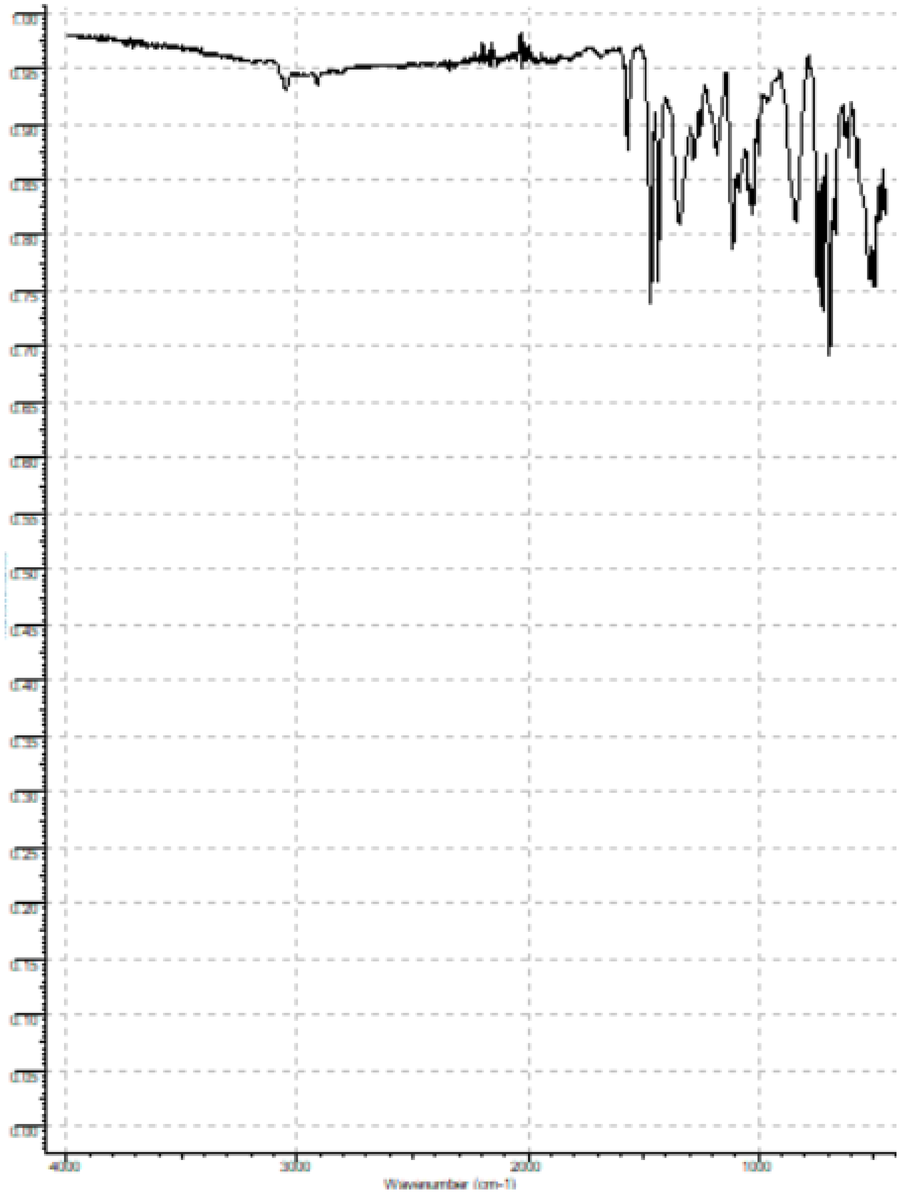

IR spectrum for **2e**

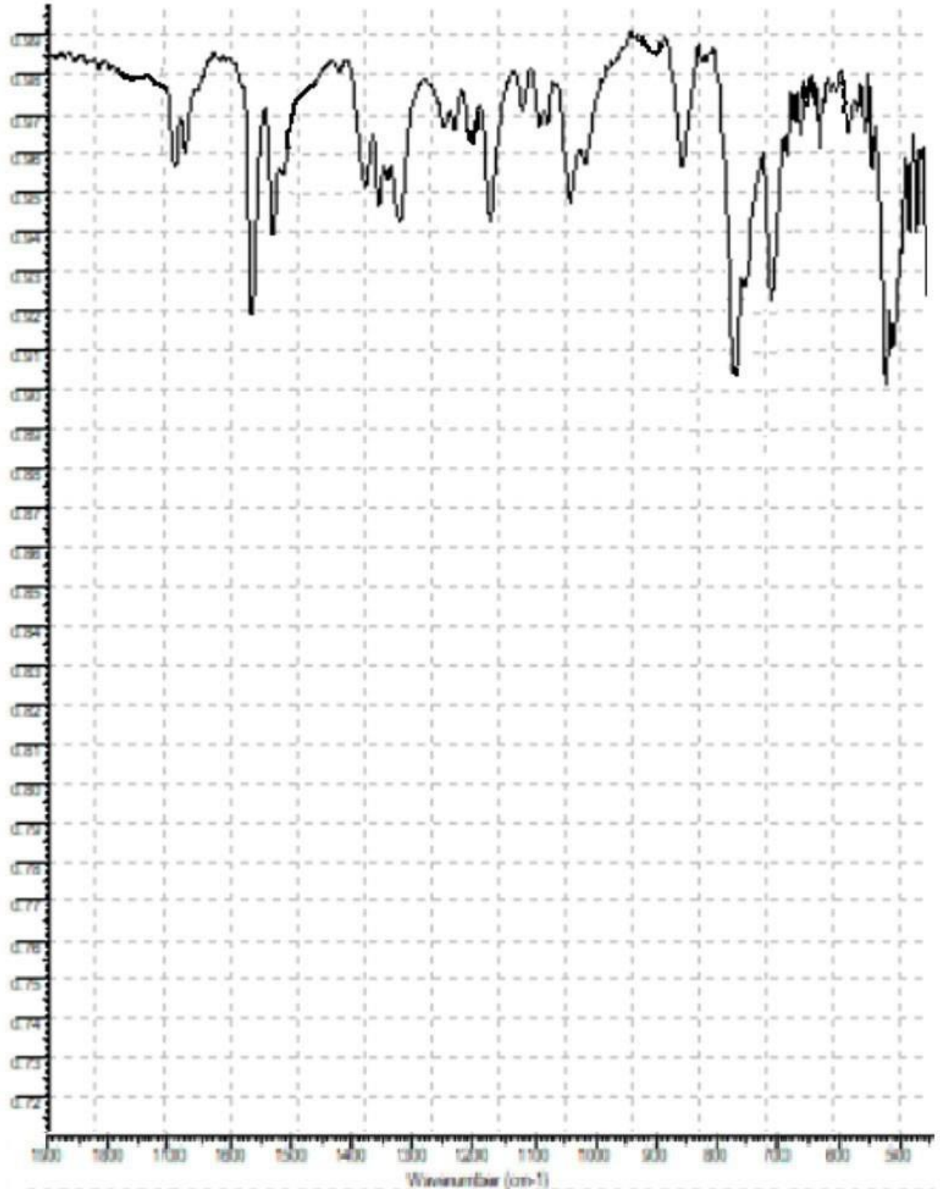

IR spectrum for **3a**

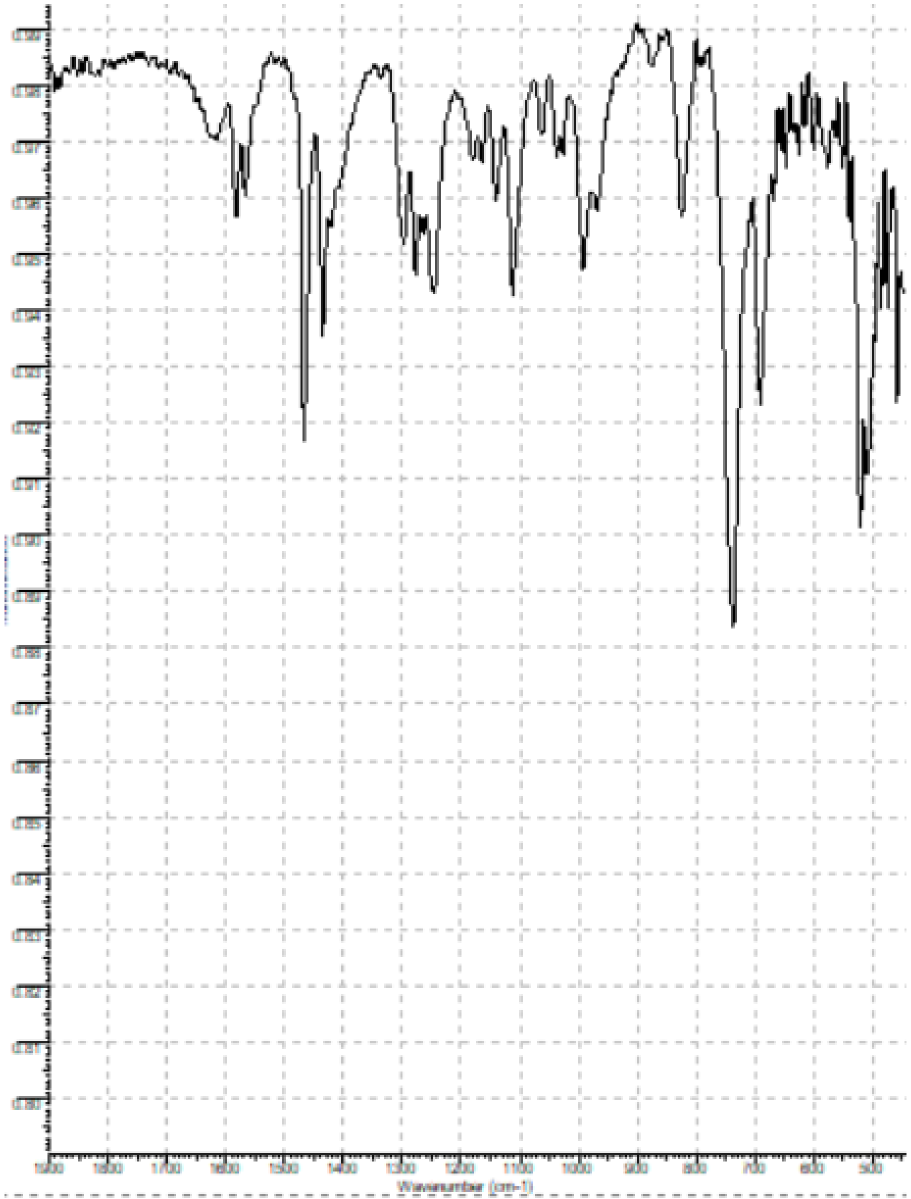

IR spectrum for **3b**

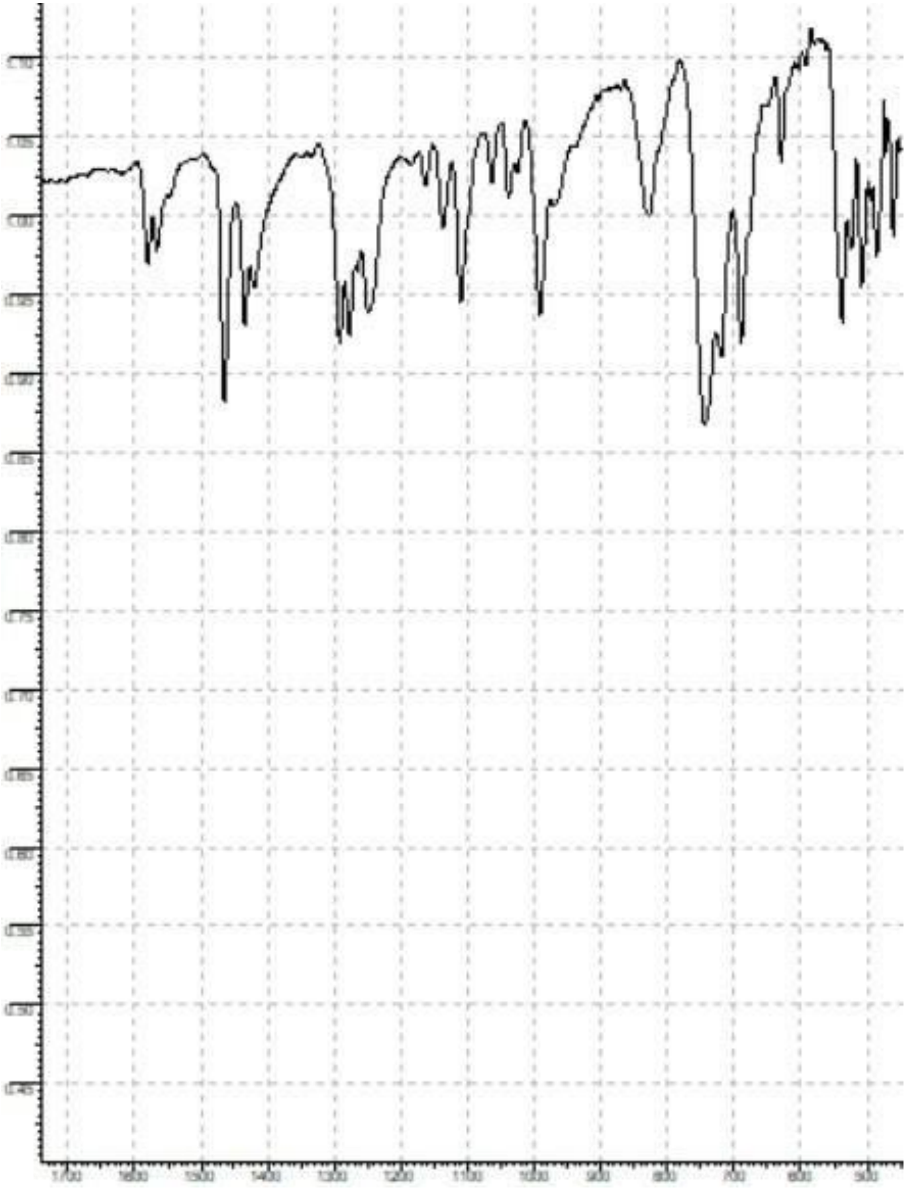

IR spectrum for **3c**

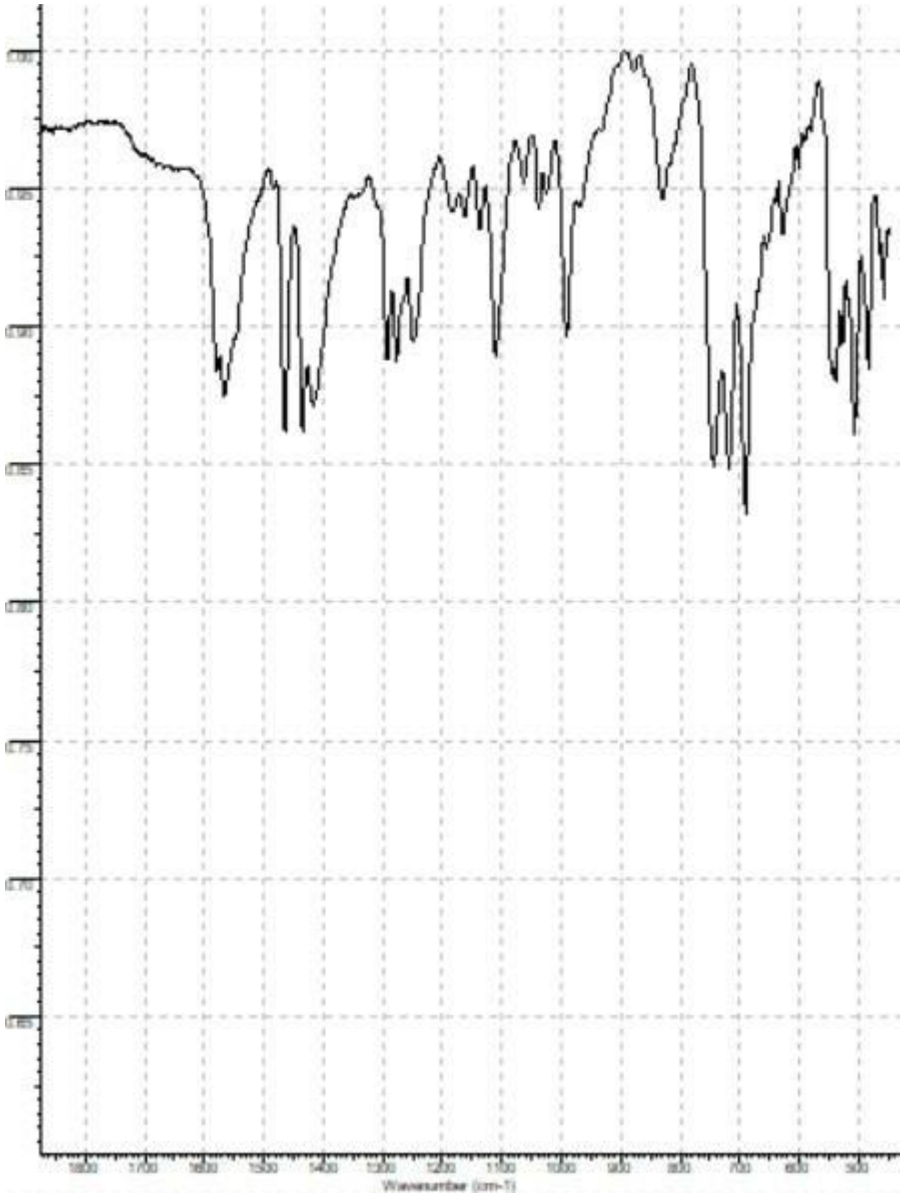

IR spectrum for **3d**

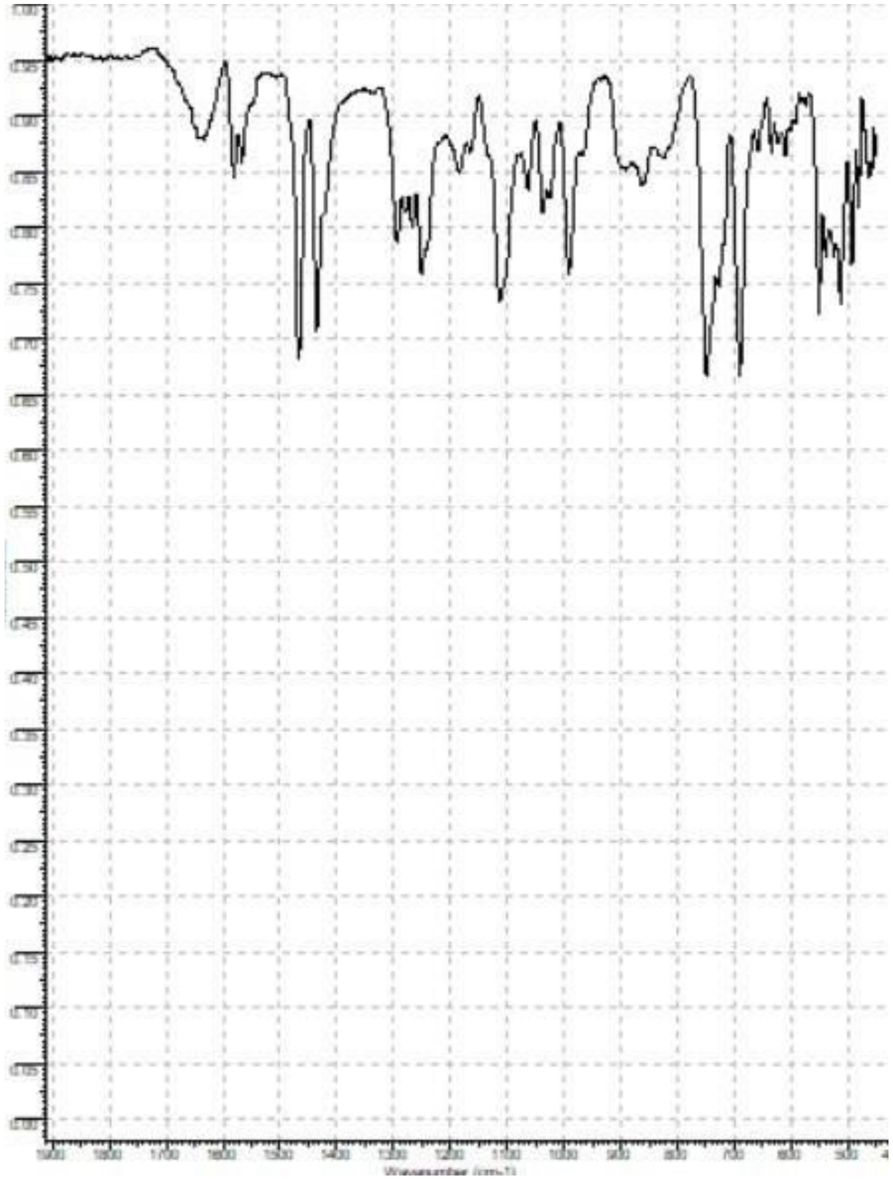

IR spectrum for **3e**

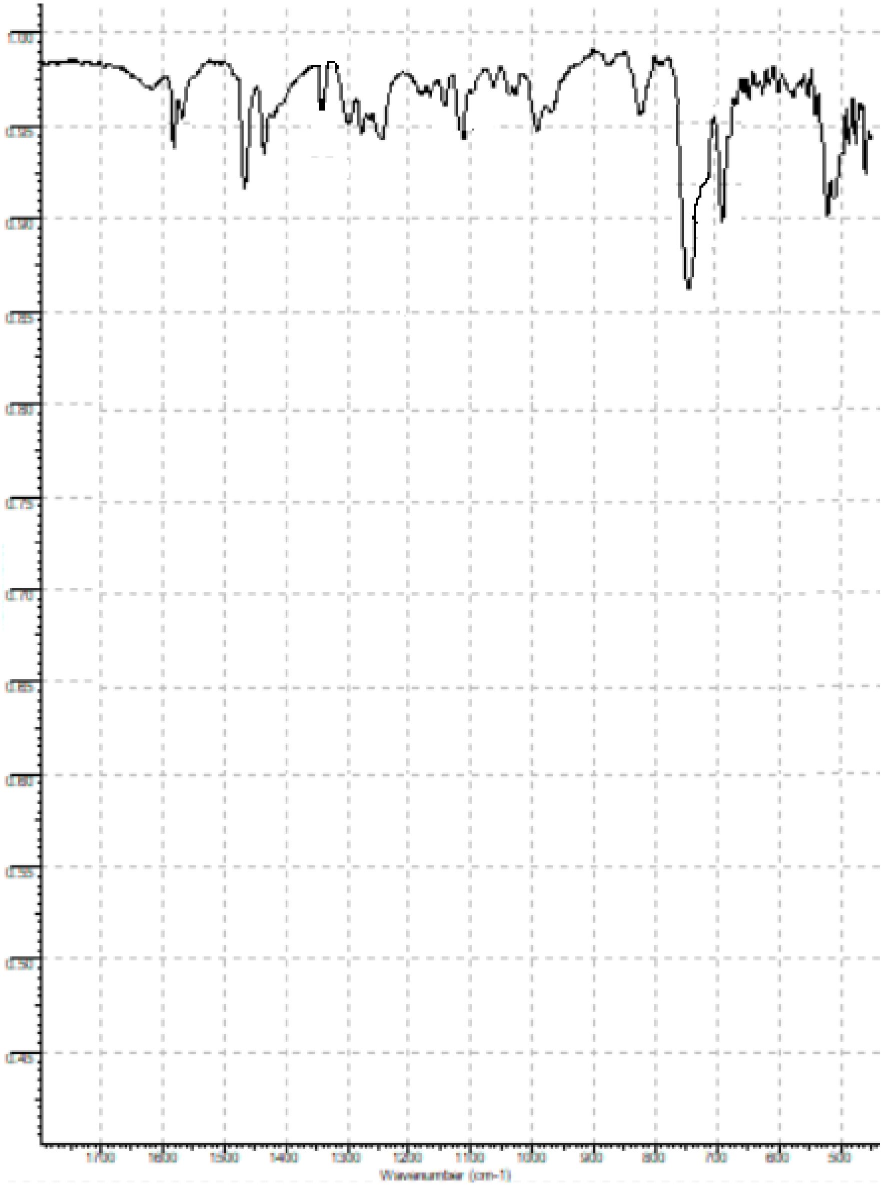

IR spectrum for **4**
